# Supplementary figures and images for: mGBP2 engages Galectin-9 for immunity against Toxoplasma gondii
Source: PLoS One. 2025 Jan 24;20(1):e0316209. doi: 10.1371/journal.pone.0316209 (PMC11761162; doi:10.1371/journal.pone.0316209)

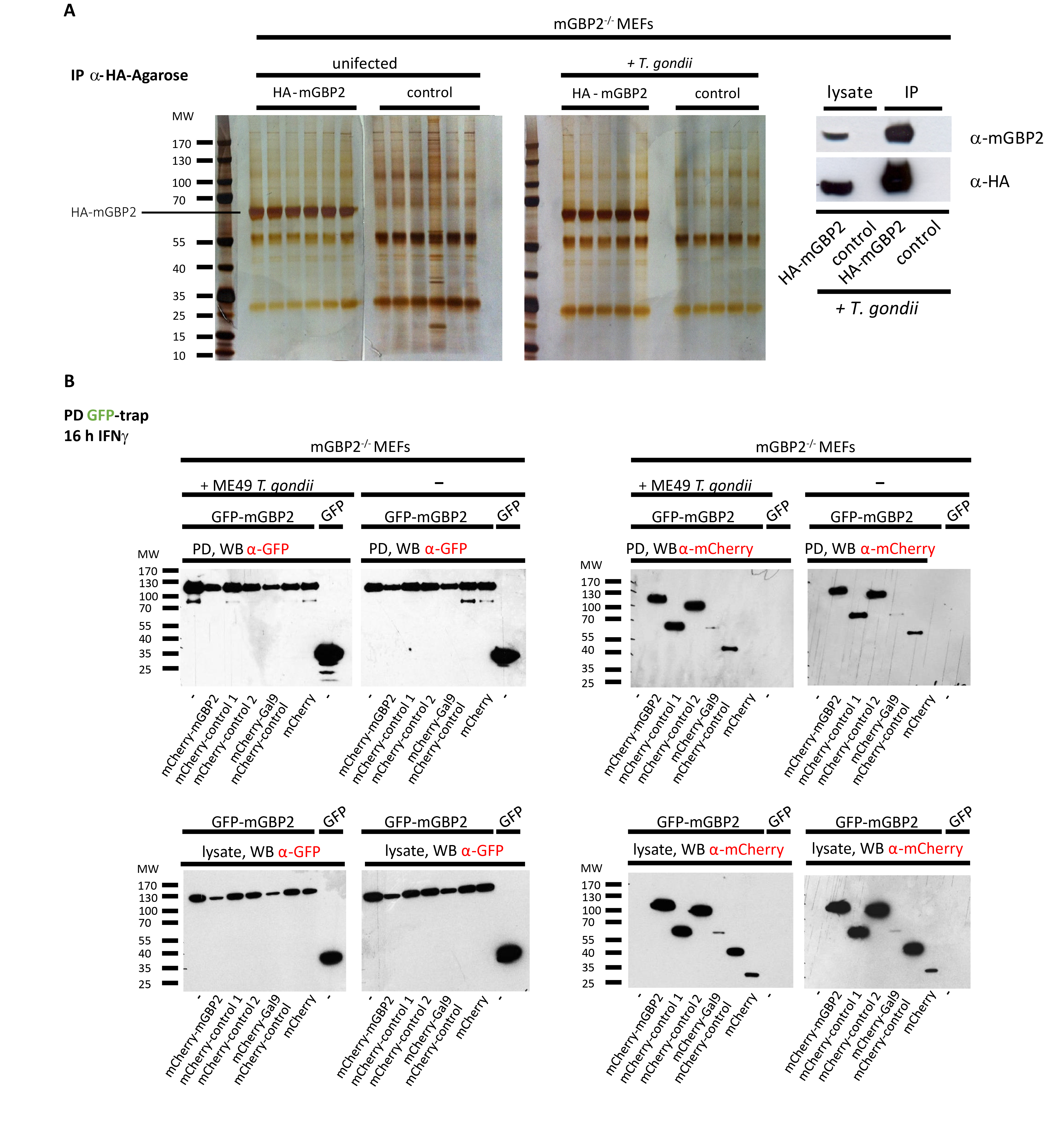

Supplement: S1 Fig — (A) mGBP2-/- MEFs were reconstituted with HA-mGBP2 or a control vector and stimulated with IFN-γ for 16 h. 1x10^6^ cells were infected for 2 h with T. gondii ME49 (MOI 50) or left uninfected. Subsequently, cells were lysed and lysate supernatants were incubated o/n with α-HA antibody coupled agarose beads at 4°C for IP. One part of IP samples was separated via 10% SDS-PAGE and labelled using silver staining. Another part of the IP samples was subjected to Western Botting and immune staining with an α-mGBP2 antiserum or an α-HA antibody. A third part of these IP samples was transferred to the MS analysis (see main text). (B) mGBP2-/- MEFs were reconstituted with GFP-mGBP2 WT as well as one of the N-terminal mCherry fusion proteins mCherry-mGBP2, or mCherry-Gal9. mCherry-Control 1, mCherry Control 2, and mCherry-Control 3 are interacting proteins that will be published elsewhere and do not relate to this study. Cells were stimulated with IFN-γ for 16 h and infected with T. gondii ME49 for 4h. Cells were lysed, and lysate supernatants were incubated o/n with GFP-Trap® beads at 4°C. Pulldown samples and appropriate cell lysate supernatants were subjected to Western Blotting. Blots were stained with α-GFP or α-mCherry antibodies. (TIF) [file pone.0316209.s001.tif]

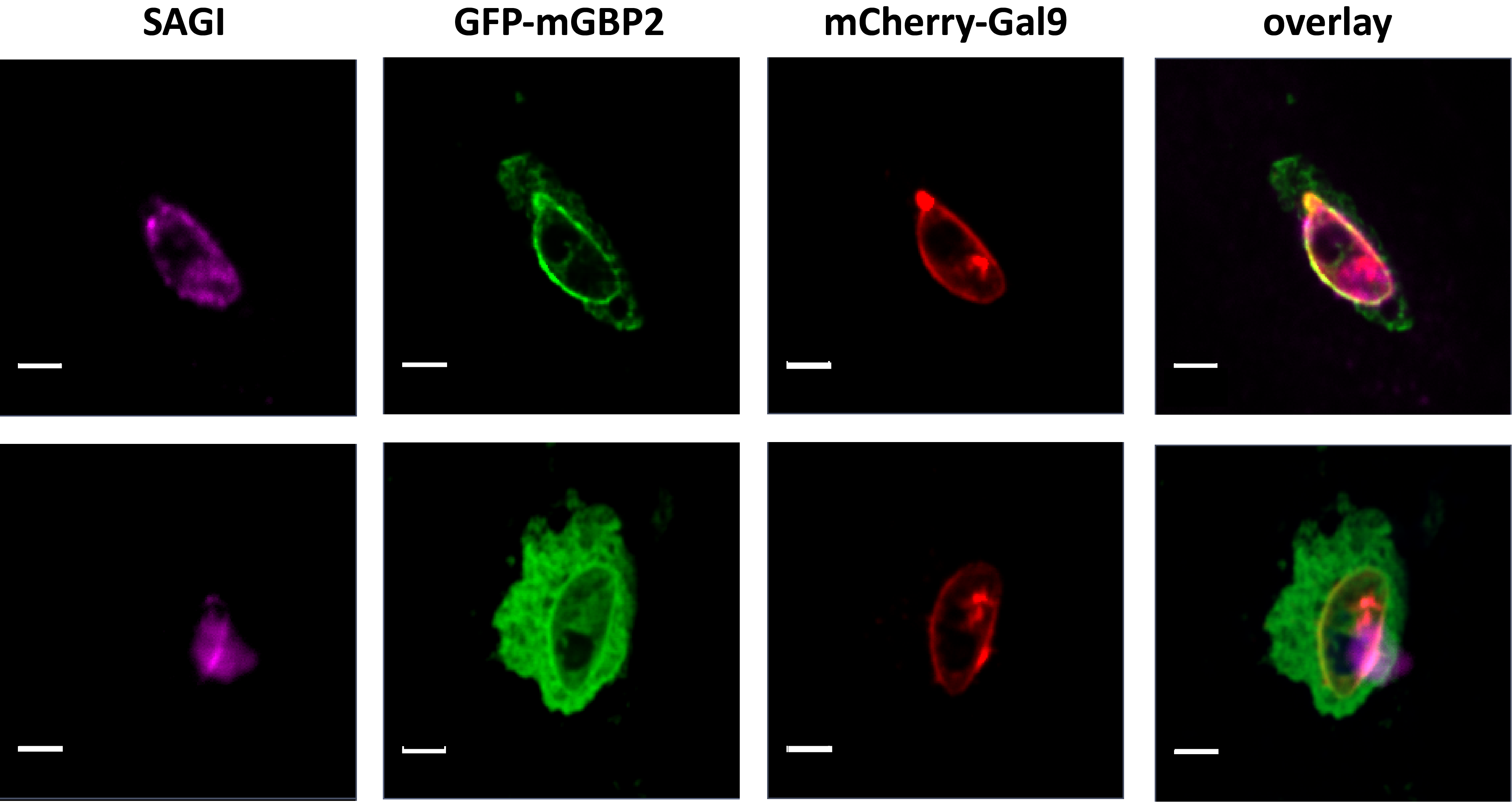

Supplement: S2 Fig — Recruitment and colocalization of mGBP2 was analyzed in GFP-mGBP2 expressing mGBP2-/- MEFs with additional transduction of either mCherry-Gal9. MEFs were stimulated with IFN-γ for 16 h and subsequently infected with T. gondii ME49 for 5 h. After fixation, T. gondii were stained with an α-SAG1 antibody and the cell nuclei were labeled with DAPI. Glass slides were analyzed by confocal microscopy. Two representative examples from three independent experiments are shown. Bars 2 μm. (TIF) [file pone.0316209.s002.tif]

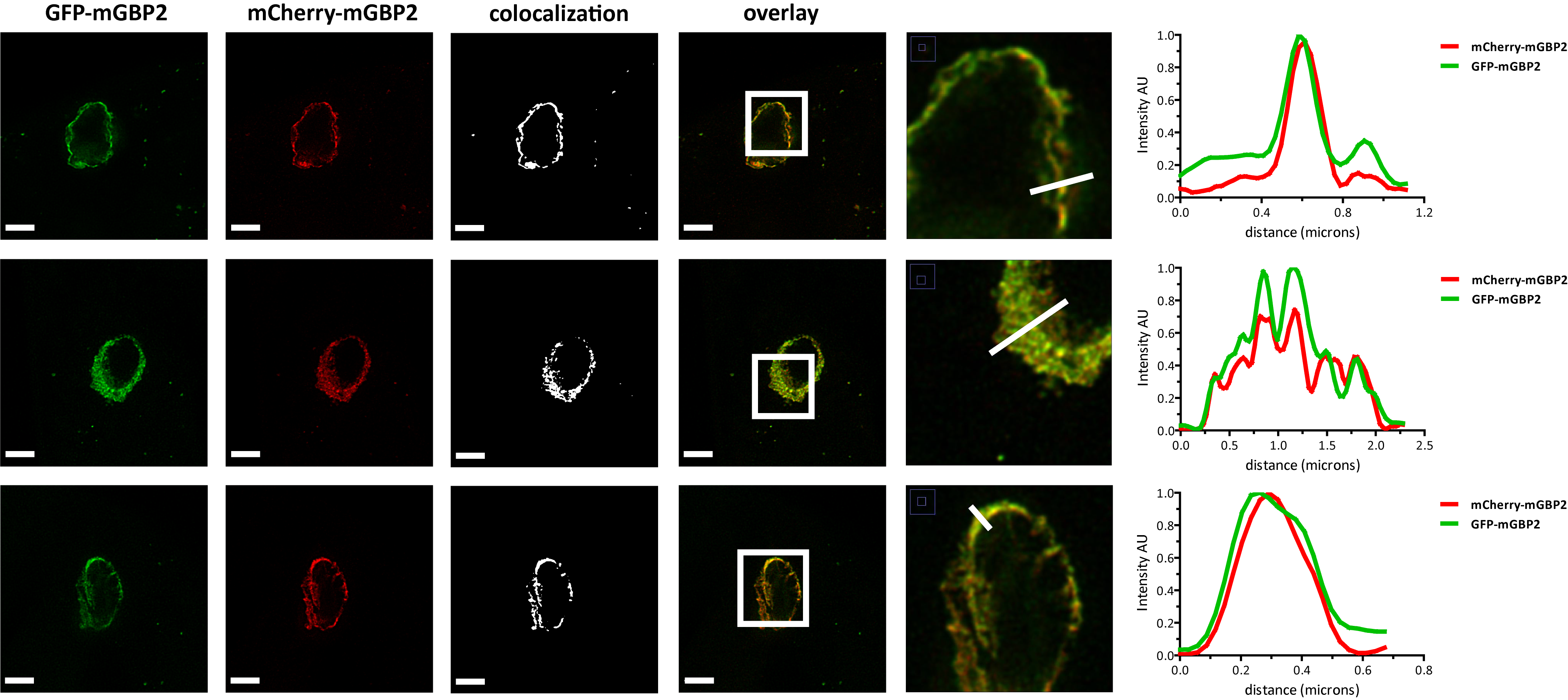

Supplement: S3 Fig — Recruitment and colocalization of mGBP2 was analyzed in GFP-mGBP2 expressing mGBP2-/- MEFs with additional transduction of mCherry-mGBP2 as control. MEFs were stimulated with IFN-γ for 16 h and subsequently infected with T. gondii ME49 for 2 h. After fixation, T. gondii were stained with an α-SAG1 antibody. Infected cells were treated with anti-RFP VHH nanobody conjugated to eGFPBoosterAtto647N and with anti-GFP VHH nanobody conjugated to eGFPBoosterAtto488 for enhancement of immunofluorescence of mCherry and GFP respectively. Glass slides were analyzed by STED microscopy. Bars 2 μm. The graphs depict a colocalization analysis of STED images with the ImageJ software (Fiji) for GFP and mCherry fluorescence. The colocalization thresholds were set to 13000-max for the Cherry and 2000-max for GFP in mCherry-mGBP2 and GFP-mGBP2 expressing cells. (TIF) [file pone.0316209.s003.tif]

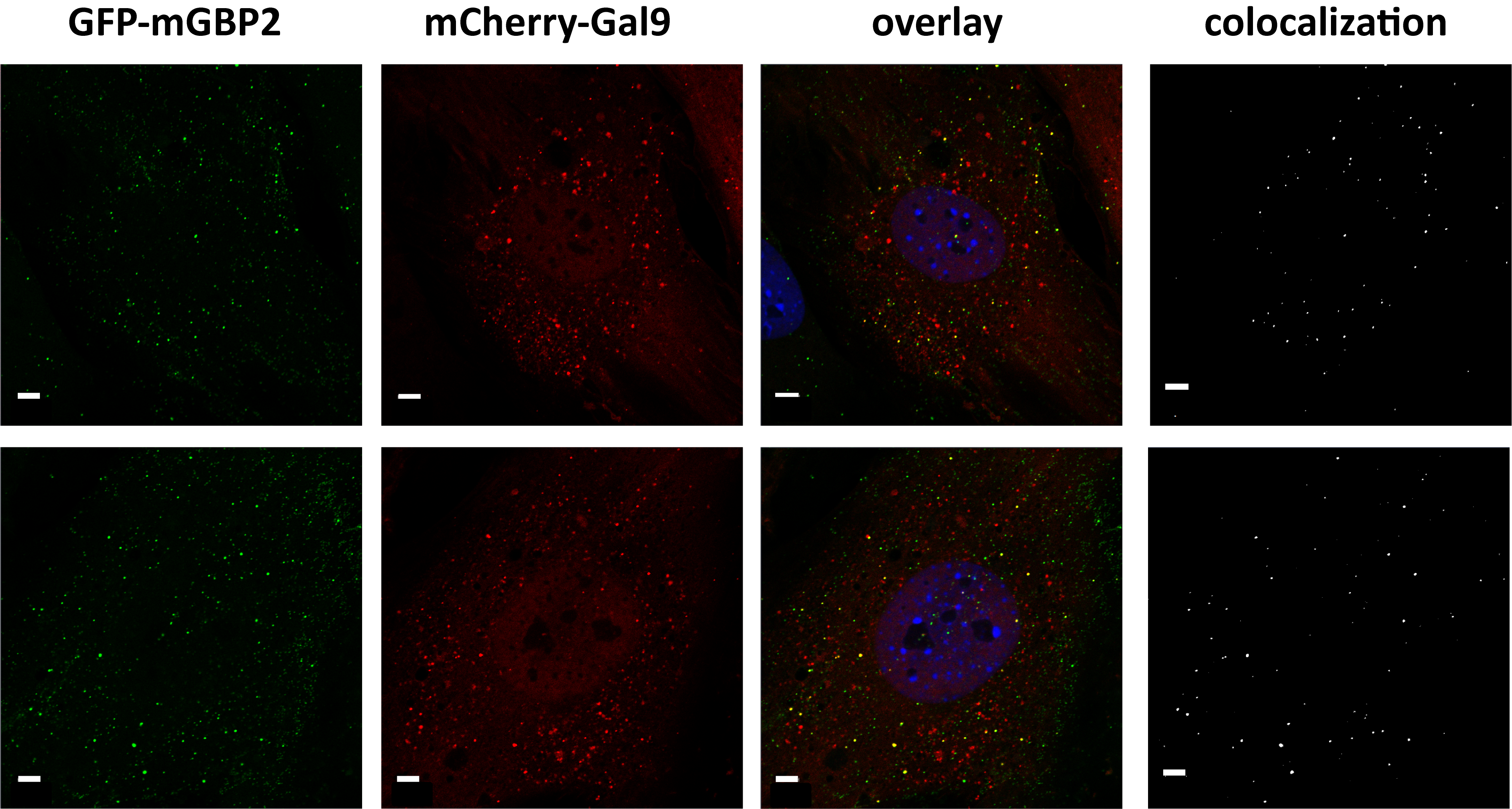

Supplement: S4 Fig — Colocalization of GFP-mGBP2 with mCherry-Gal9 was analyzed in mGBP2-/- MEFs reconstituted with the indicated fusion proteins. Cells were stimulated with IFN-γ for 16 h. After fixation, cell nuclei were labeled with DAPI. Glass slides were analyzed by confocal microscopy. Two representative examples from three independent experiments are shown. Bars 5 μm. The right column depicts the results for a colocalization analysis using the Image Visualization and Analysis Software (Imaris) for GFP and mCherry fluorescence. (TIF) [file pone.0316209.s004.tif]

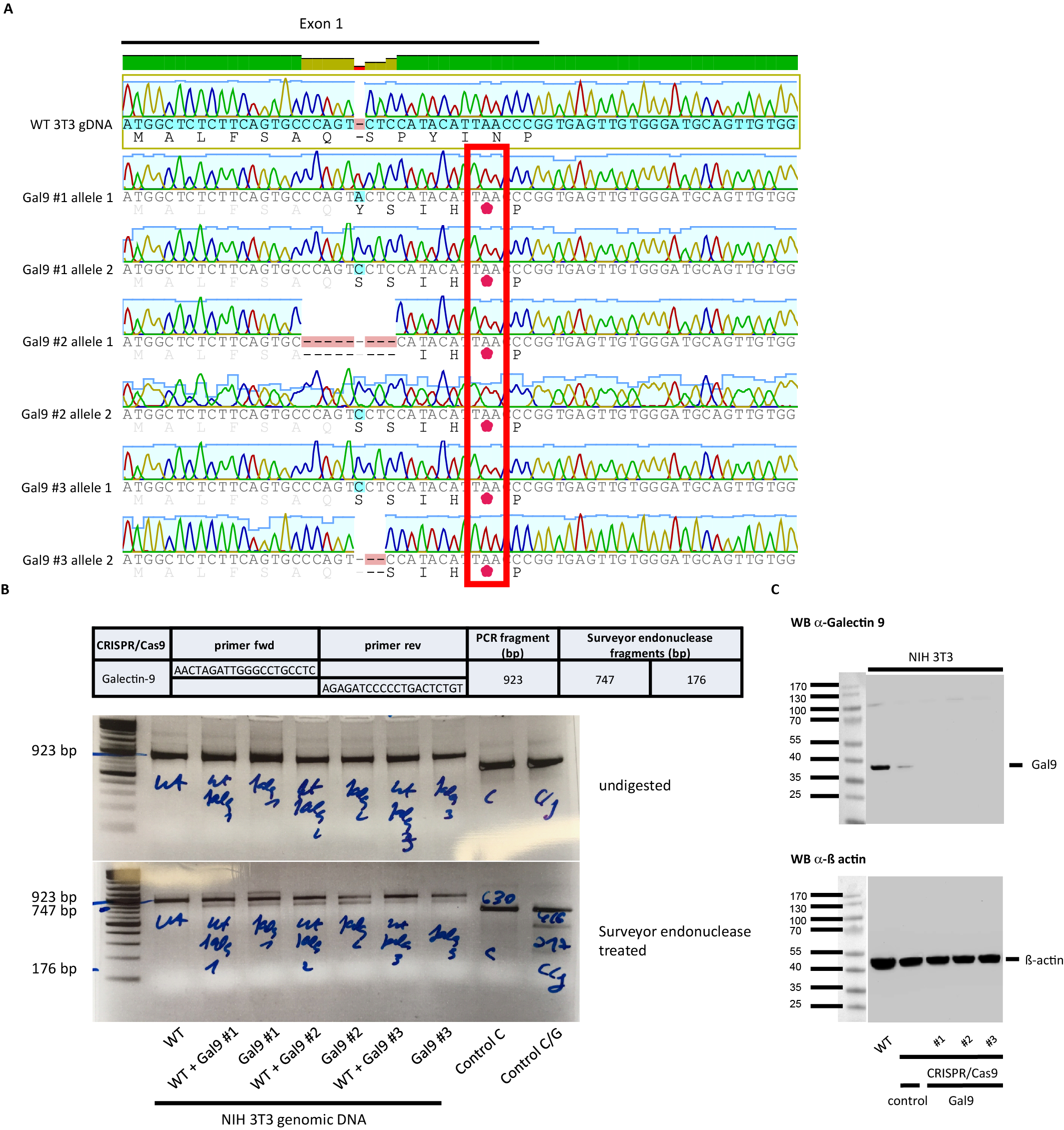

Supplement: S5 Fig — (A) CRISPR/Cas9 and sgRNA specific for Gal9 were expressed in NIH 3T3 fibroblasts. Clones were picked and DNA from WT and three CRIPR/Cas9 Gal9 targeted cell clones of NIH 3T3 fibroblasts was isolated, PCR amplified (923 bp). PCR products were cloned into the pCR2.1 TA-cloning vector. The Gal9 mutations were verified by Sanger sequencing. Indels, AA sequence and premature stop codons (*) are indicated. (B) For independent mutation analysis, the Surveyor® Mutation Detection Kit for Standard Gel Electrophoresis was employed. Here, a mismatch‐specific DNA endonuclease to scan mutations and polymorphisms in heteroduplex DNA is used. PCR amplicons from Gal9 mutant NIH 3T3 clones (test) and WT (reference) DNA were hybridized and the mixtures of hetero‐ and homo‐duplexes were submitted to Surveyor Nuclease digestion. The reference DNA alone, treated similarly, served as a negative control. DNA fragments were analyzed by agarose gel electrophoresis. The formation of new cleavage products, due to the presence of one or more mismatches, is indicated by the presence of additional bands. The relative size of these cleavage products indicates the location of the mismatch or mismatches. (C) Lysate supernatants of NIH 3T3 cells from different CRISPR/Cas9 Gal9 sgRNA targeted clones and from WT cells were analyzed by WB. Cells were stimulated with IFN-γ for 16 h. Blots were stained with α-Gal9 antibodies. (TIF) [file pone.0316209.s005.tif]

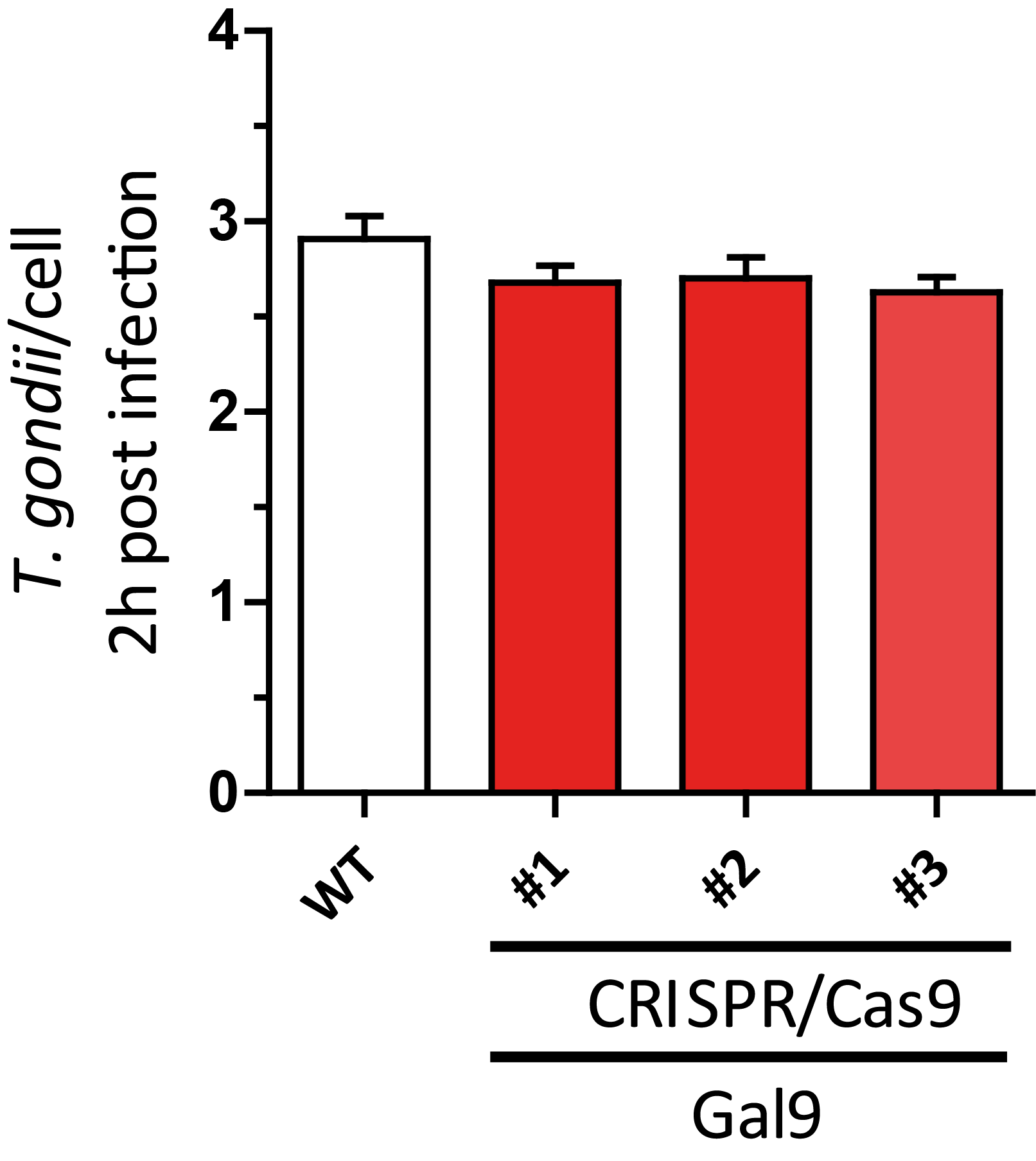

Supplement: S6 Fig — The infection rates of T. gondii ME49 were analyzed in WT and independent NIH 3T3 cell line clones with verified Gal9 inactivation. Cells were stimulated with IFN-γ for 16 h and subsequently infected with T. gondii ME49. After fixation, T. gondii were stained with the α-SAGI antibody and the cell nuclei were labeled with DAPI. Glass slides were analyzed by confocal microscopy. Bars, 5 μm. The amounts of parasites per cell were quantified 2 h after infection. (TIF) [file pone.0316209.s006.tif]

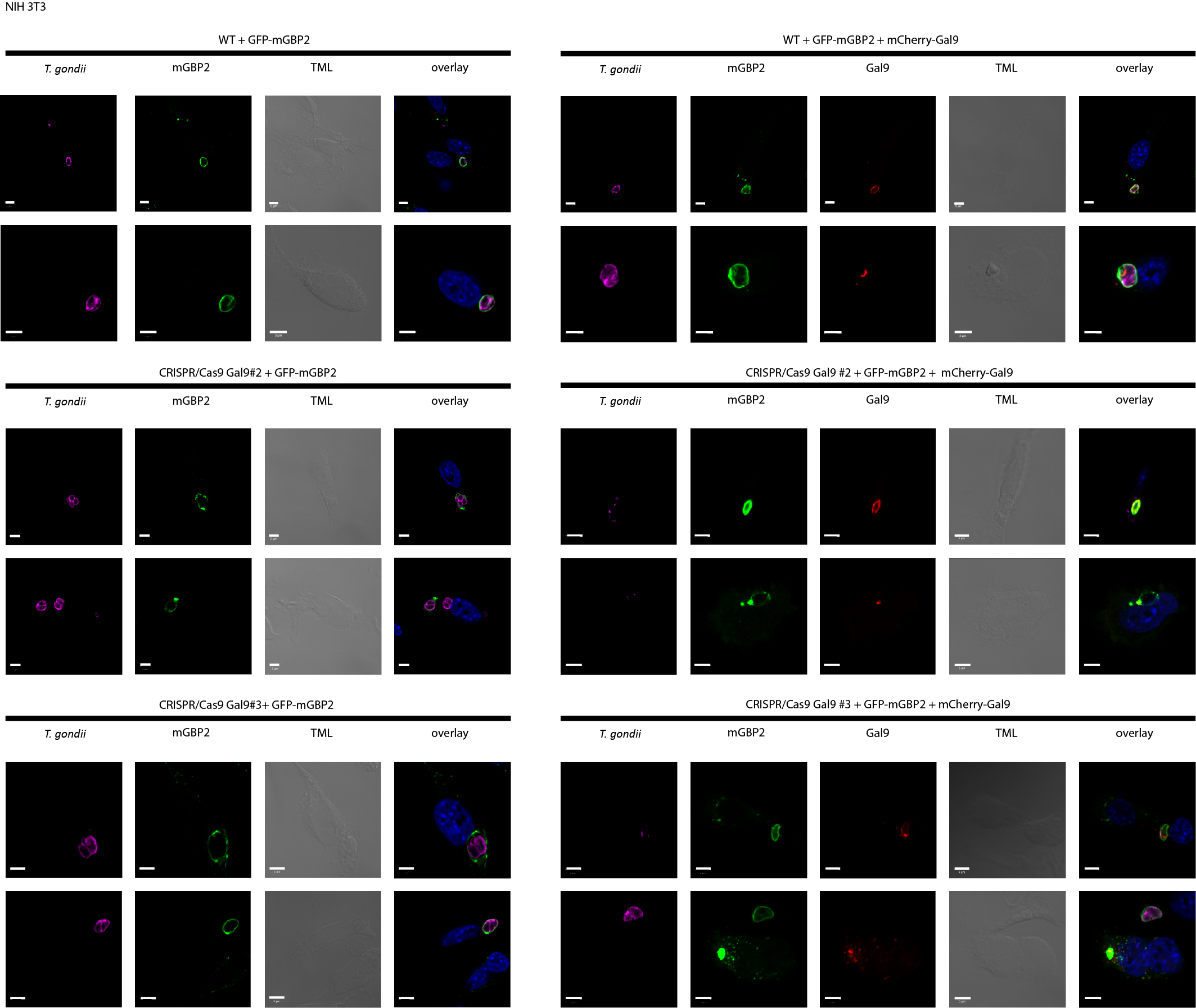

Supplement: S7 Fig — WT and independent NIH 3T3 cell line clones with verified CRISPR/Cas9 mediated inactivation of Gal9 (see main text and S5 Fig) were stimulated with IFN-γ for 16 h and subsequently infected with T. gondii ME49 for 24 h. After fixation, T. gondii were stained with an α-SAGI antibody and the cell nuclei were labeled with DAPI. Glass slides were analyzed by confocal microscopy. TML = transmitted light. Bars, 5 μm. (TIF) [file pone.0316209.s007.tif]

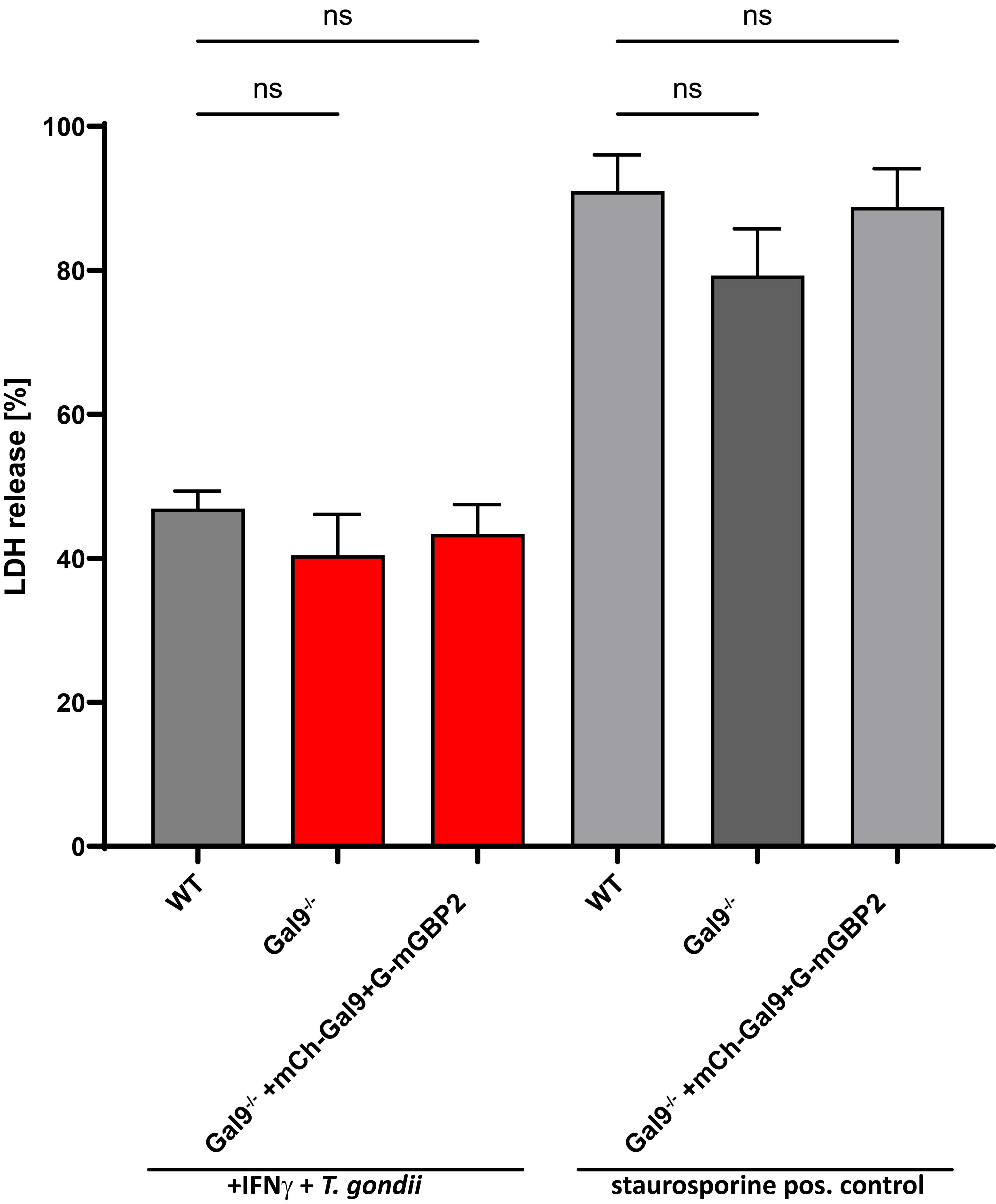

Supplement: S8 Fig — WT and independent NIH 3T3 cell line clones with verified CRISPR/Cas9 mediated inactivation of Gal9 (see main text and S5 Fig) and NIH 3T3 cell line clones with verified CRISPR/Cas9 mediated inactivation of Gal9 reconstituted with mCherry-Gal9 were stimulated with IFN-γ for 16 h and subsequently infected with T. gondii ME49 for 24 h. Subsequently, an LDH release assay was performed. Positive controls were obtained by treating the cells with 1mM Staurosporine for 24h. Shown are three independent experiments, which were respectively performed in technical triplicates to minimize pipetting errors. Plotted are means +/- SD. Statistical analysis was performed using one-way ANOVA followed by Dunnett’s multiple comparison test.; *: p < = 0.05. Only statistically significant comparisons were labelled. (TIF) [file pone.0316209.s008.tif]
