# Supplementary material for: mGBP2 engages Galectin-9 for immunity against Toxoplasma gondii
Source: PLoS One. 2025 Jan 24;20(1):e0316209. doi: 10.1371/journal.pone.0316209 (PMC11761162; doi:10.1371/journal.pone.0316209)

Fig. 2A upper panel

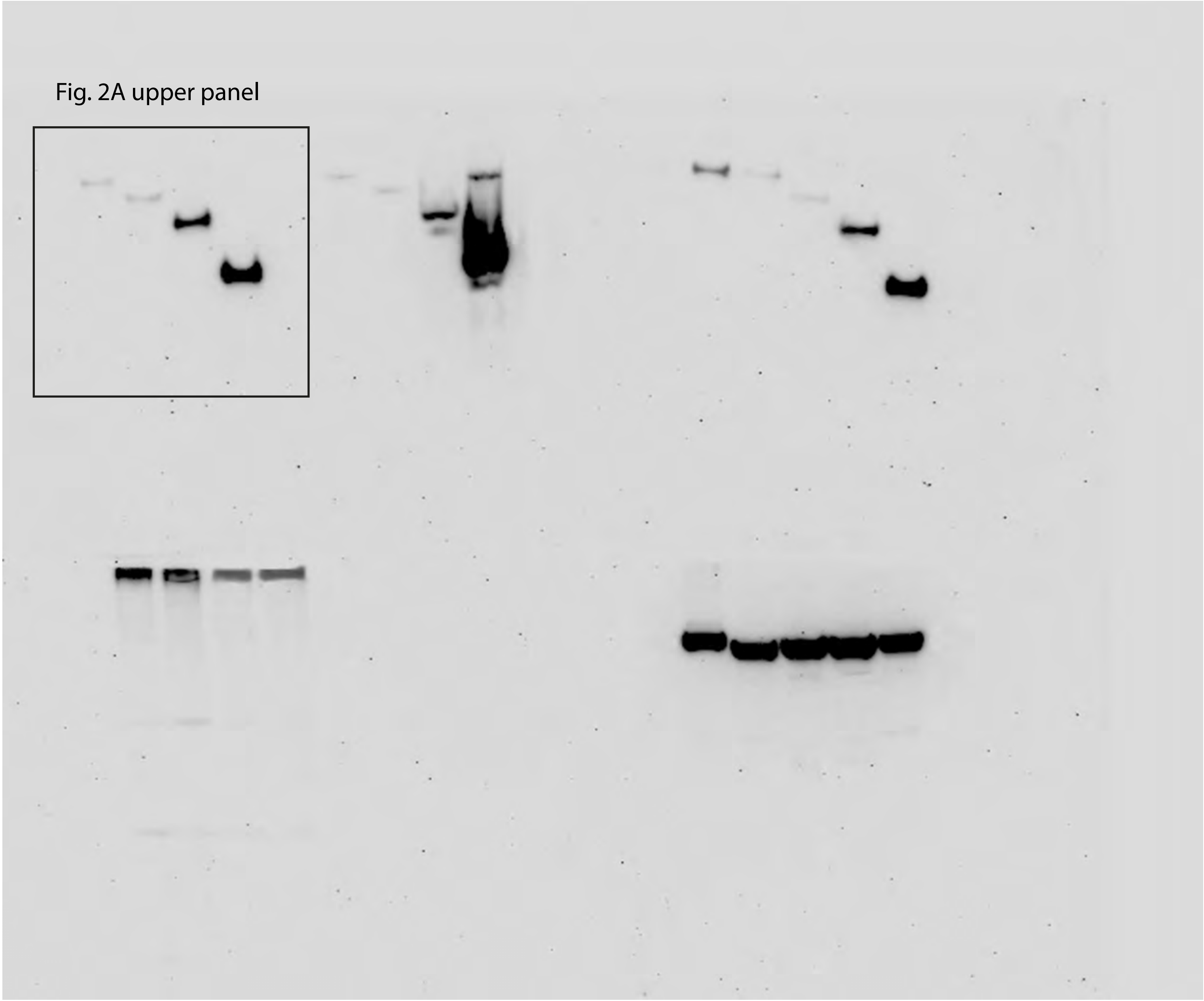

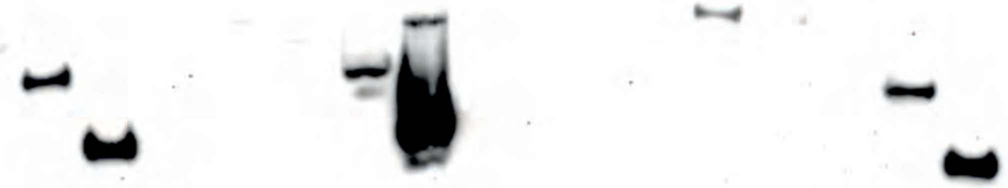

Fig. 2B lower panel

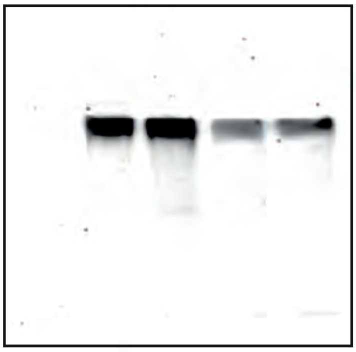

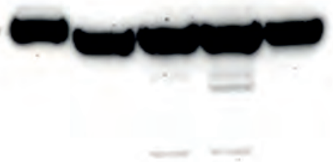

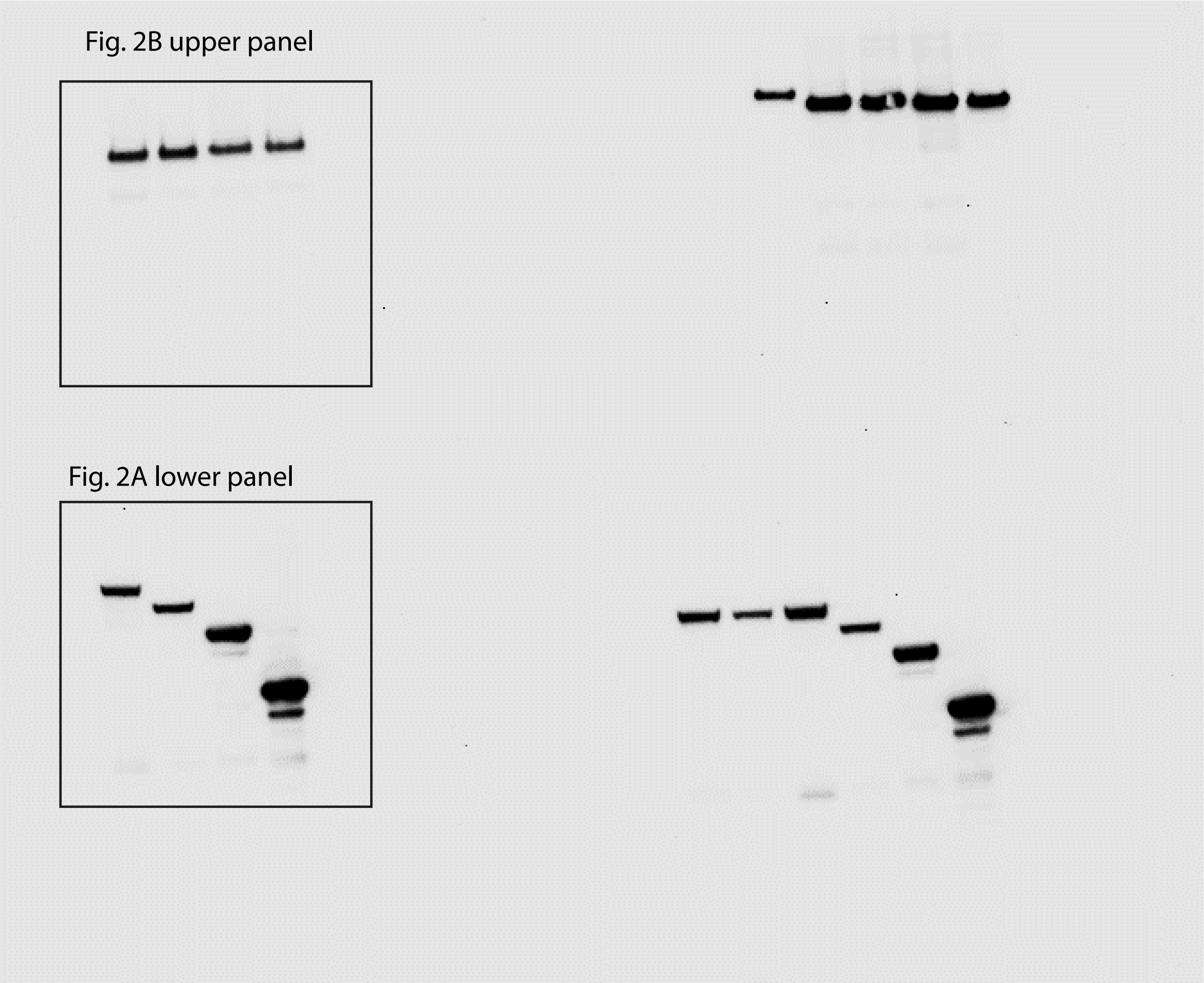
Fig. 2B upper panel
Fig. 2A lower panel

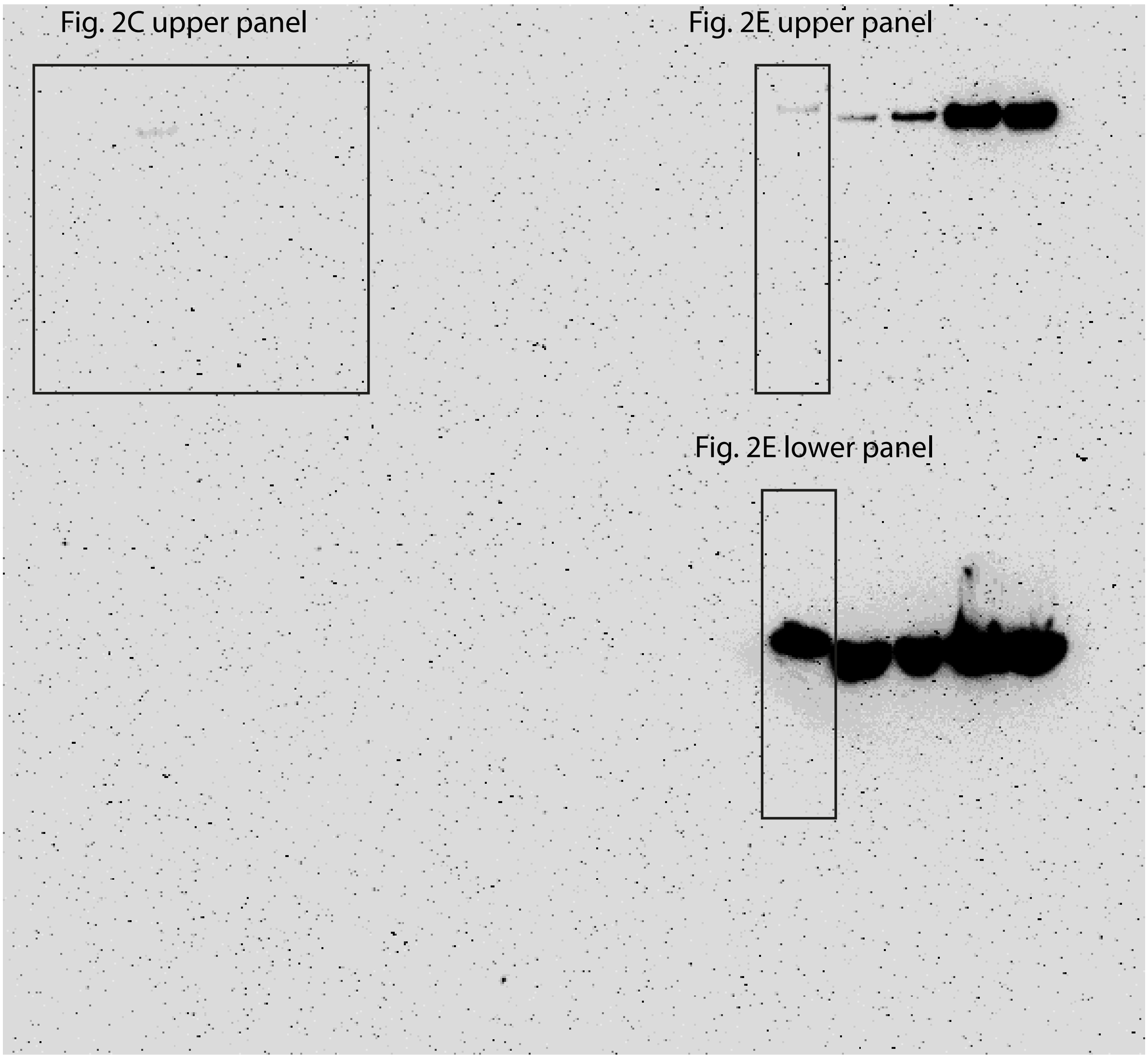
Fig. 2C upper panel
Fig. 2E upper panel
Fig. 2E lower panel

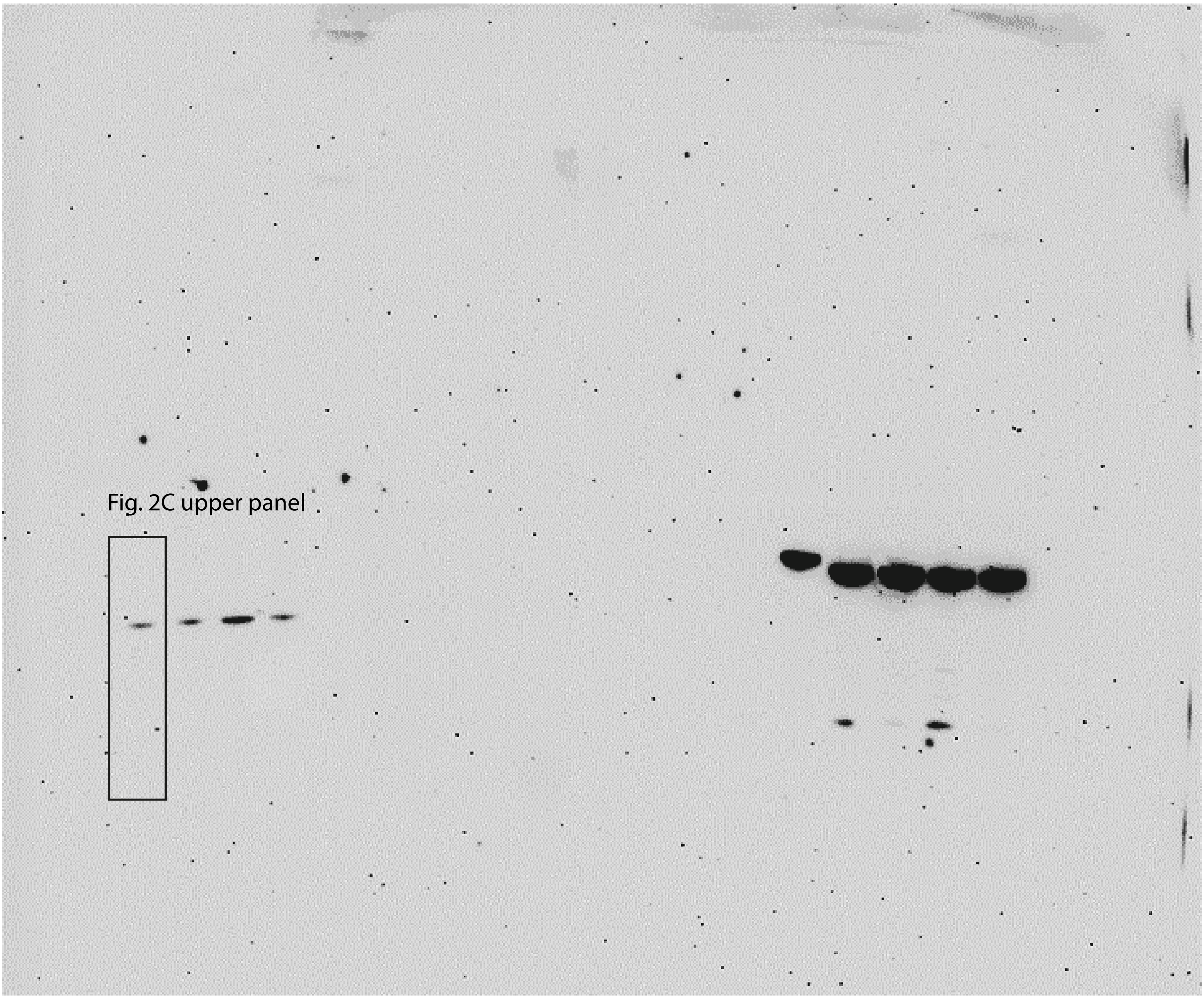
Fig. 2C upper panel

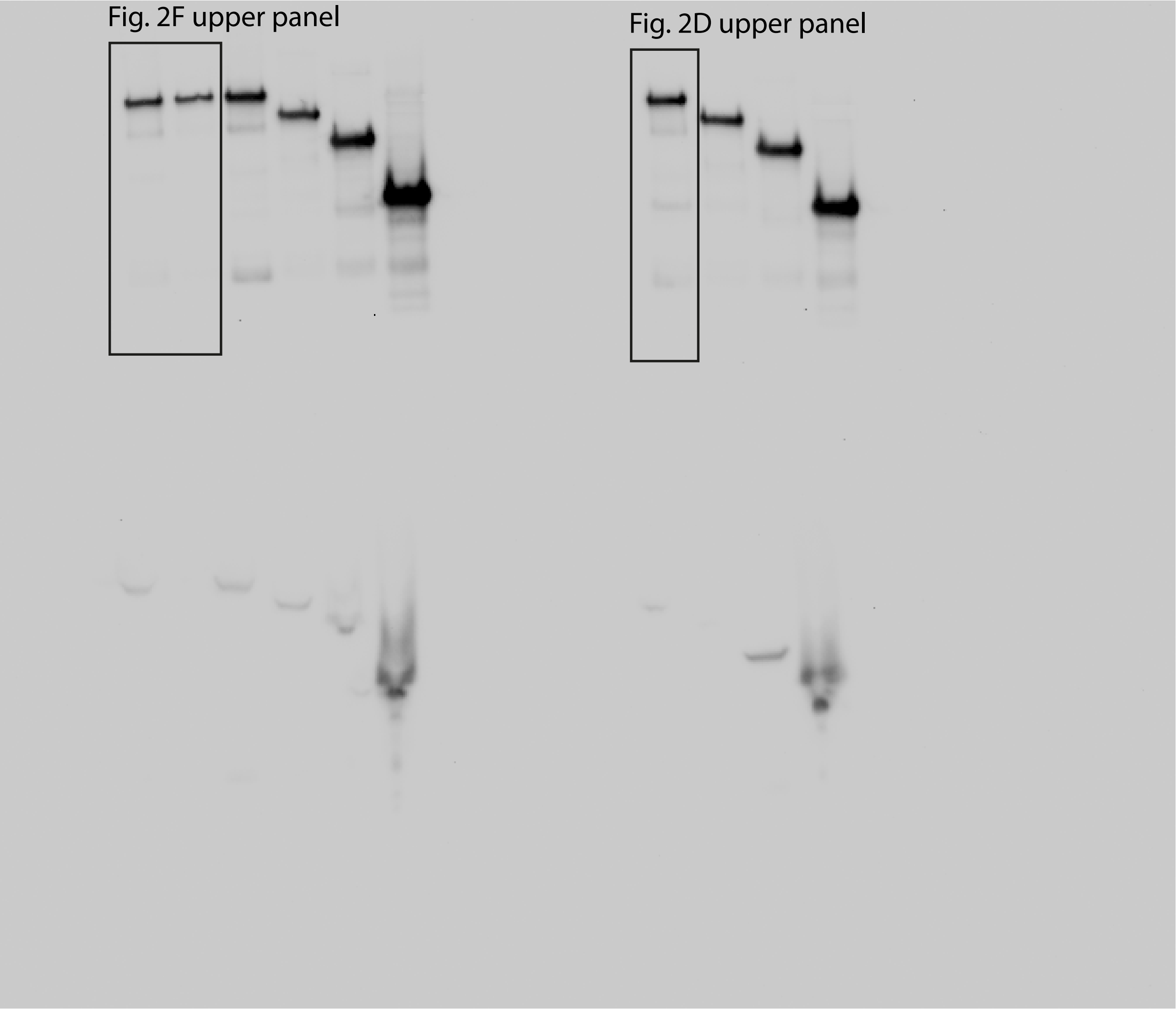
Fig. 2F upper panel
Fig. 2D upper panel

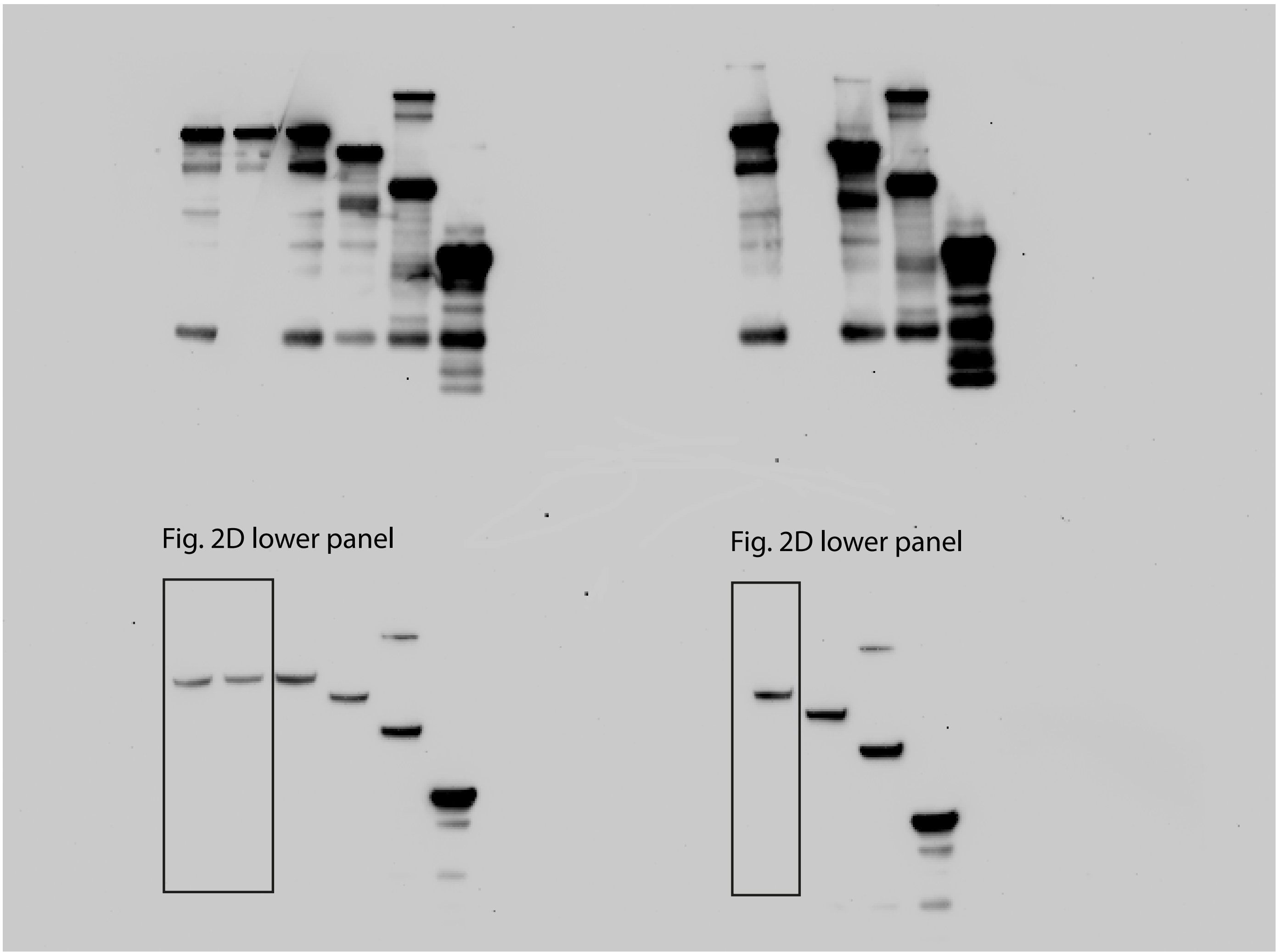
Fig. 2D lower panel
Fig. 2D lower panel

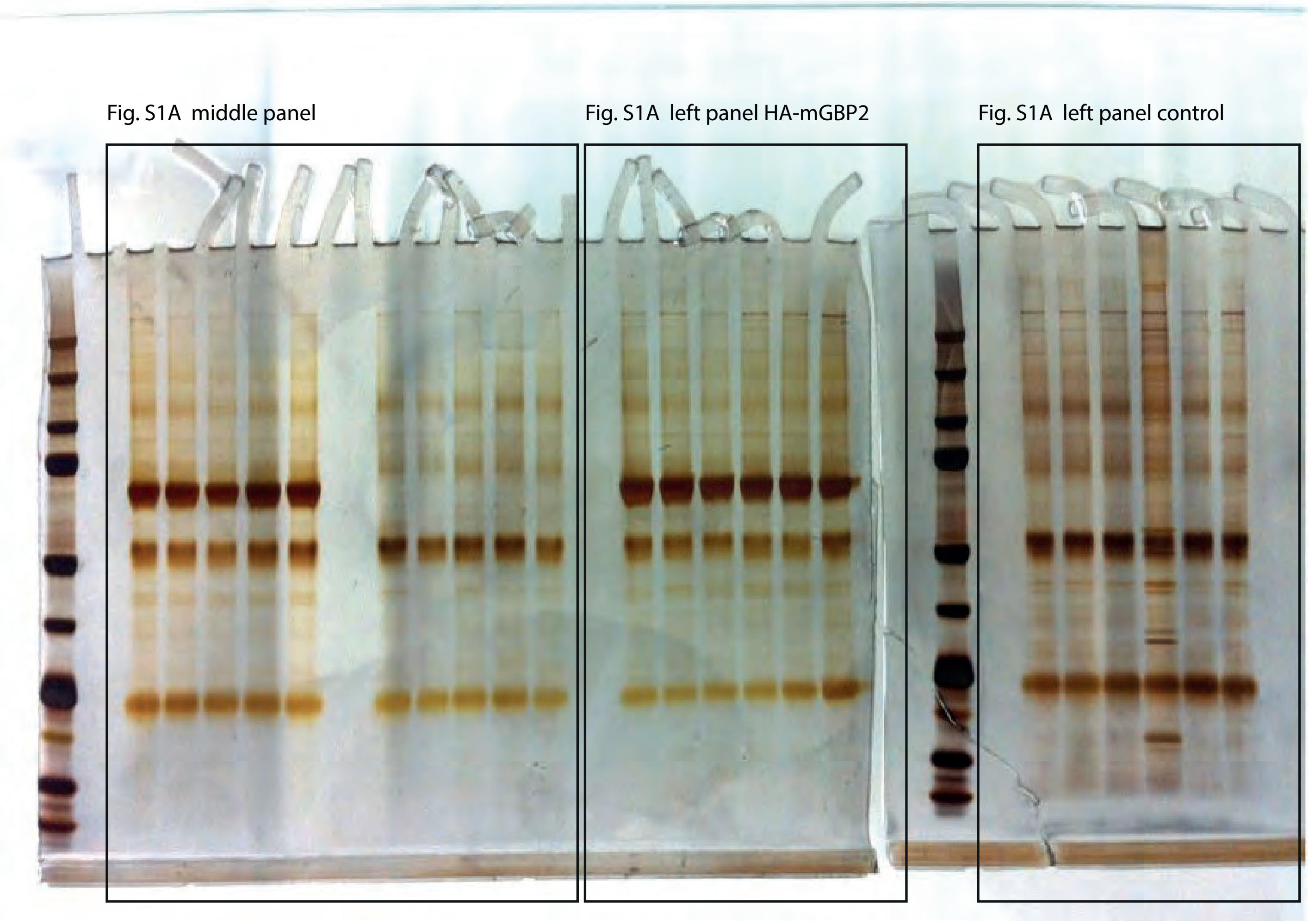
Fig. S1A middle panel
Fig. S1A left panel HA-mGBP2
Fig. S1A left panel control

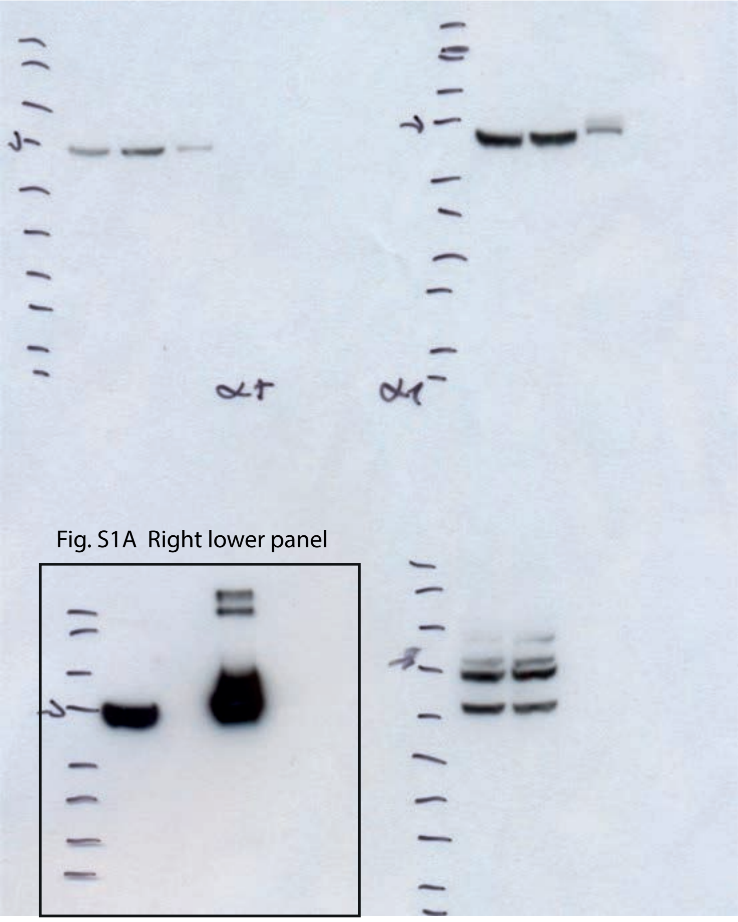
α5
α1
Fig. S1A Right lower panel

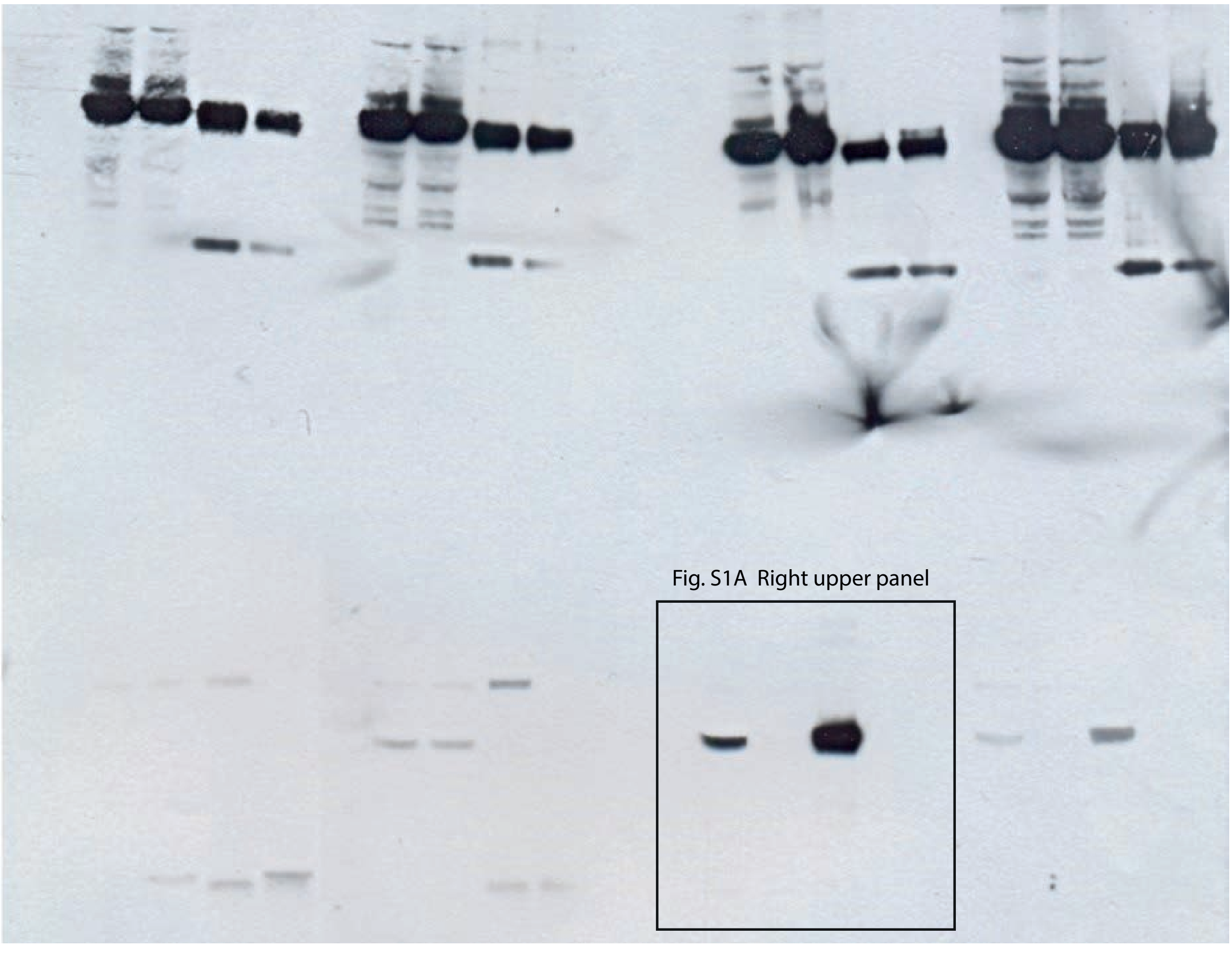
Fig. S1A Right upper panel

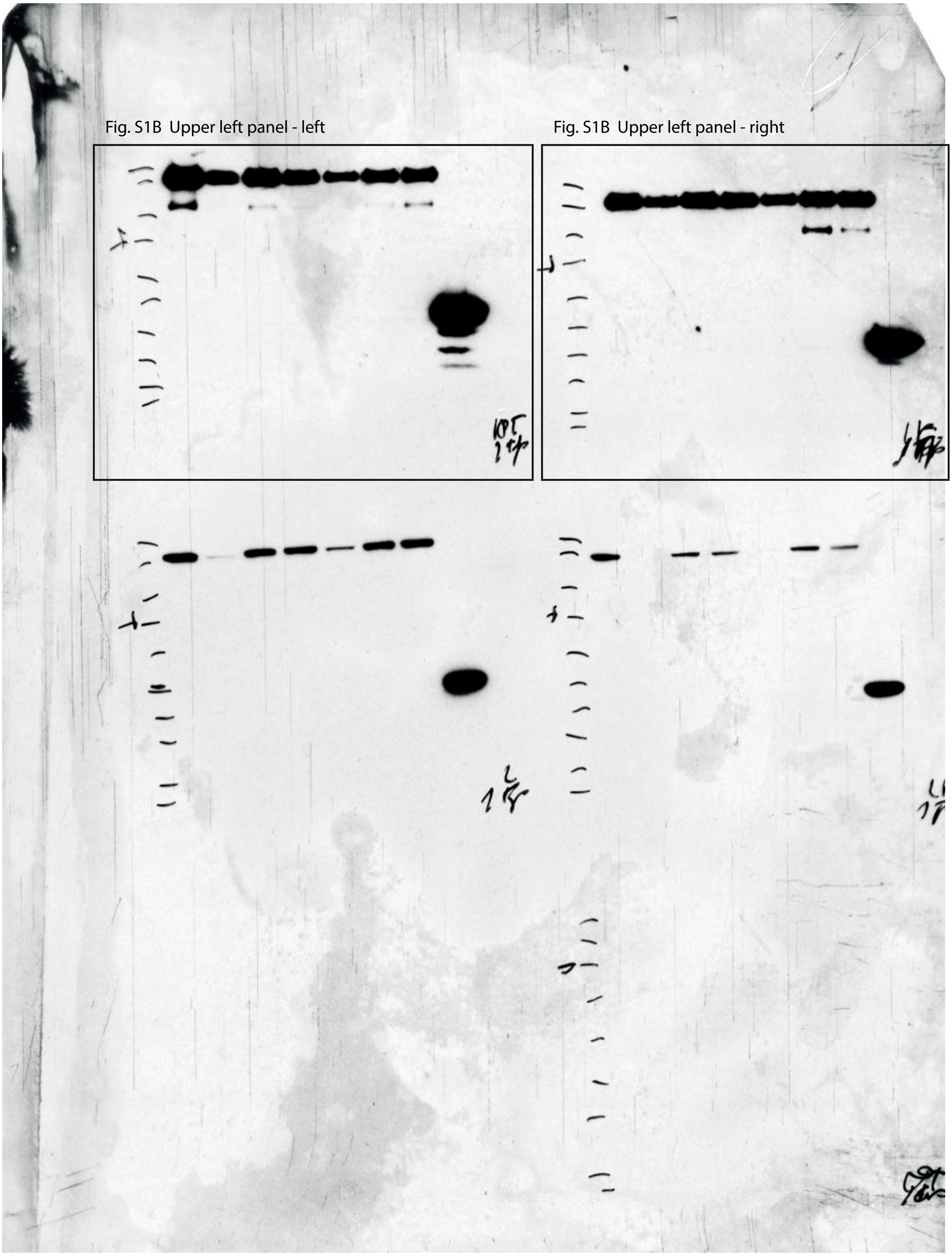

Fig. S1B Upper left panel - left

Fig. S1B Upper left panel - right

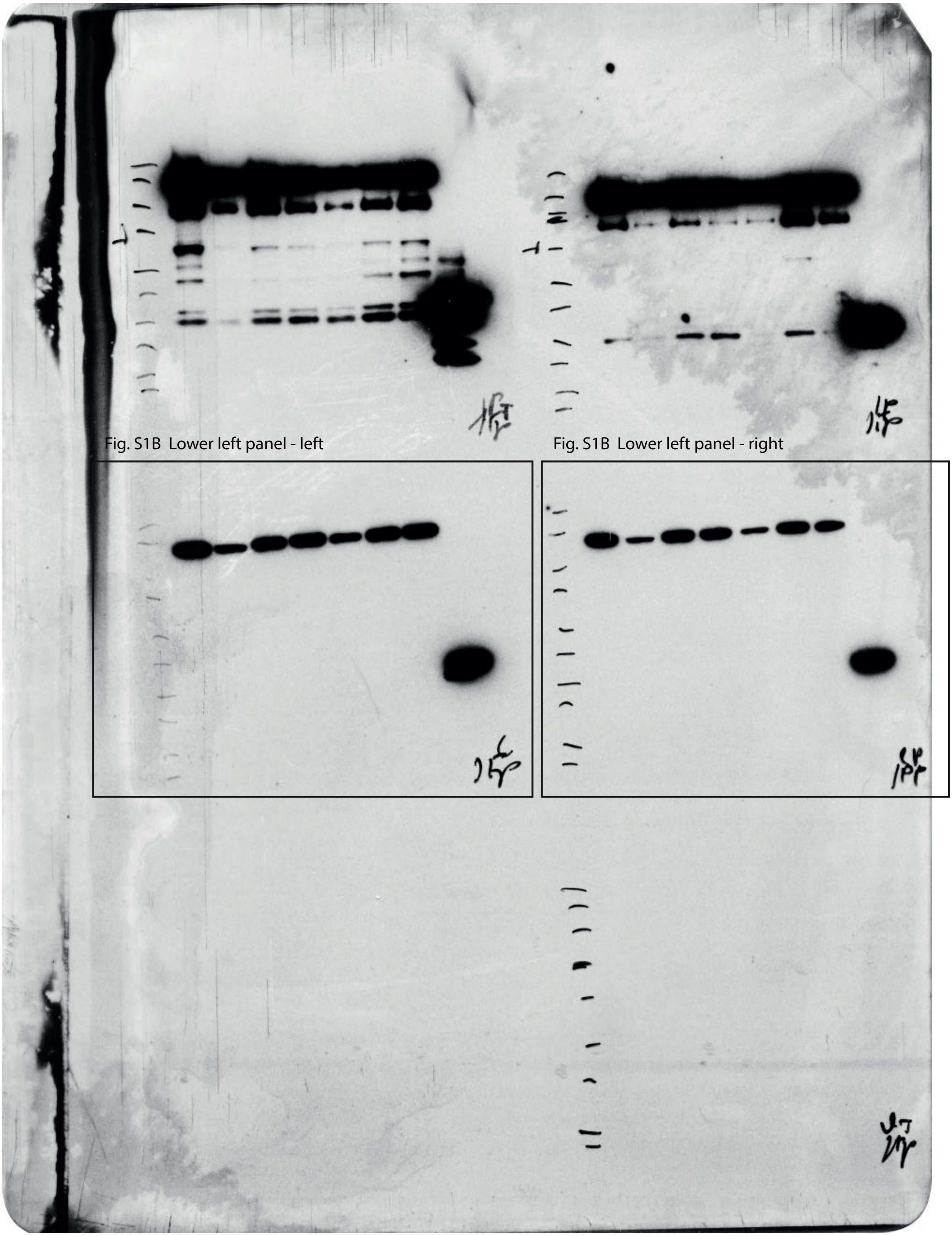

Fig. S1B Lower left panel - left

Fig. S1B Lower left panel - right

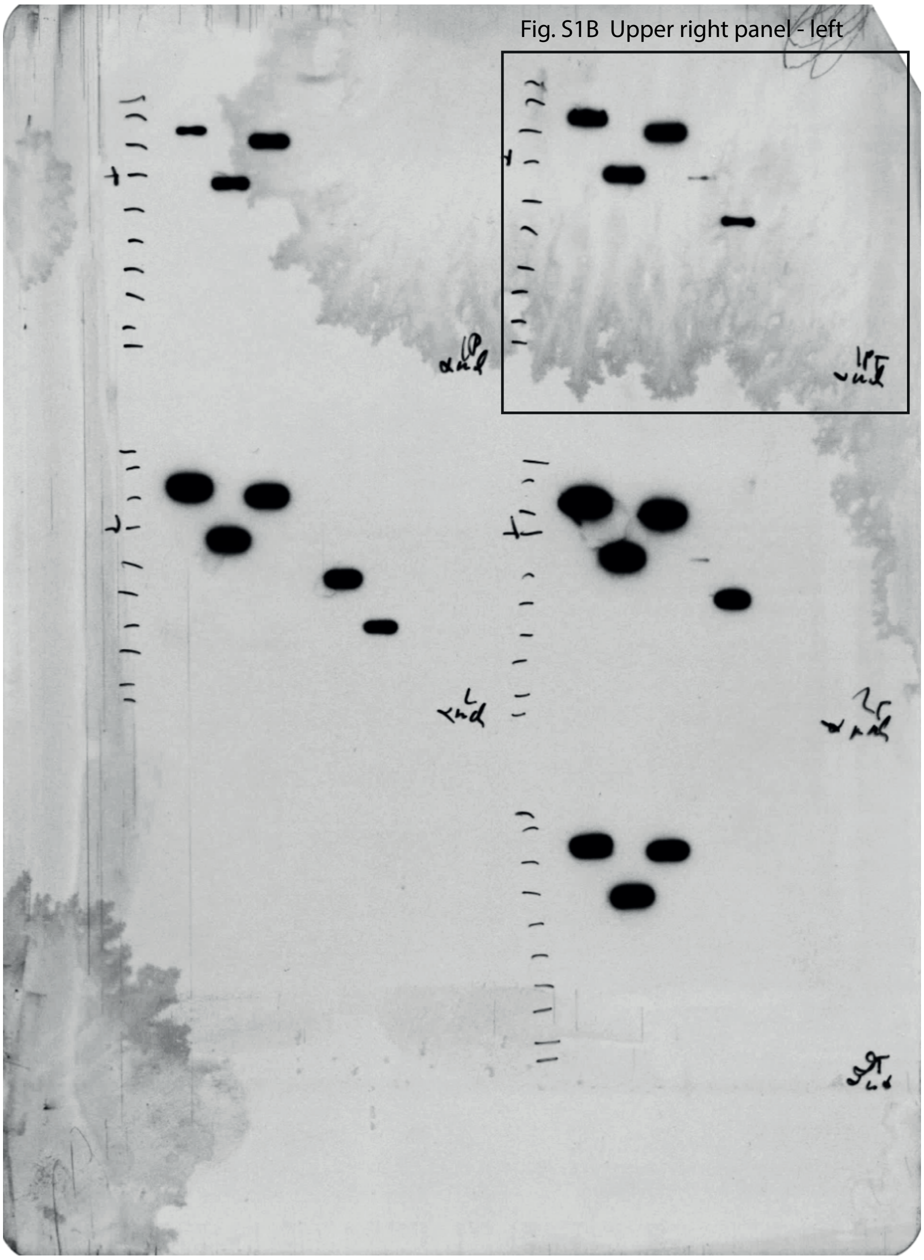

Fig. S1B Upper right panel - left

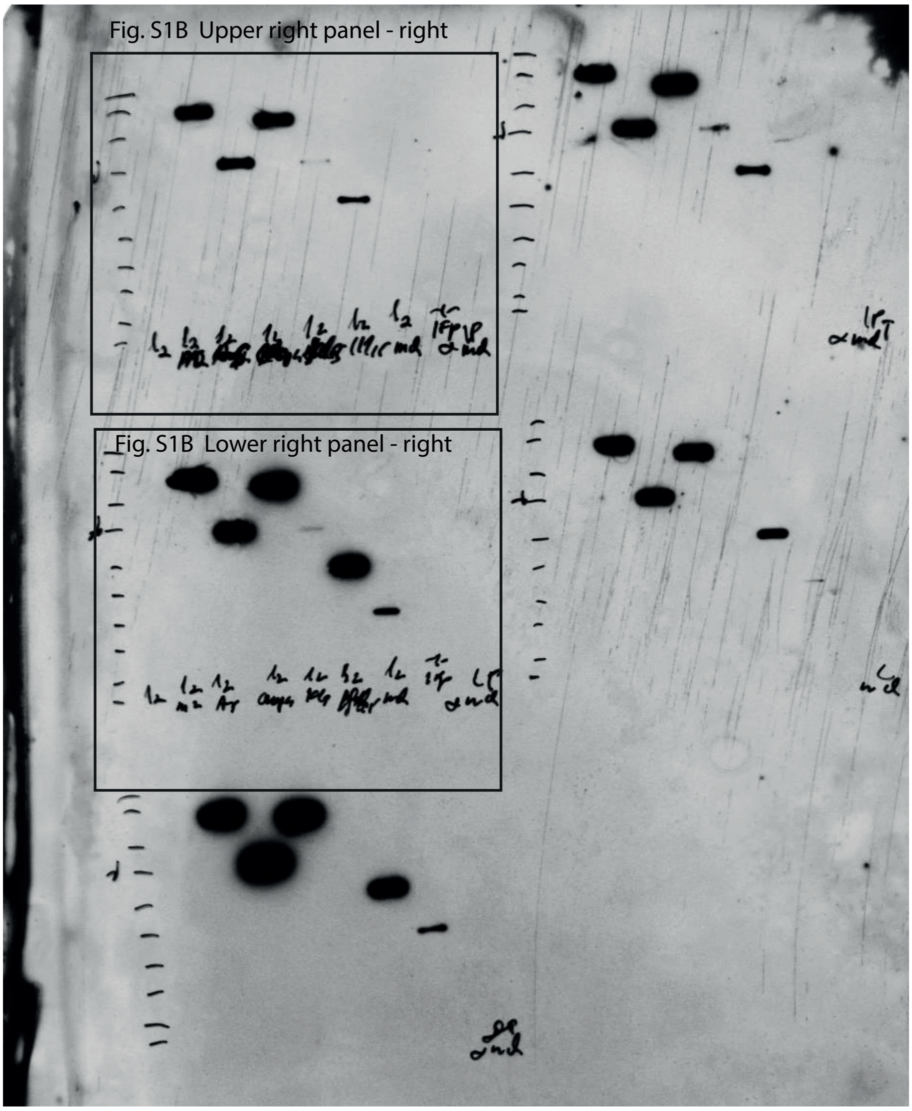
Fig. S1B Upper right panel - right
Fig. S1B Lower right panel - right

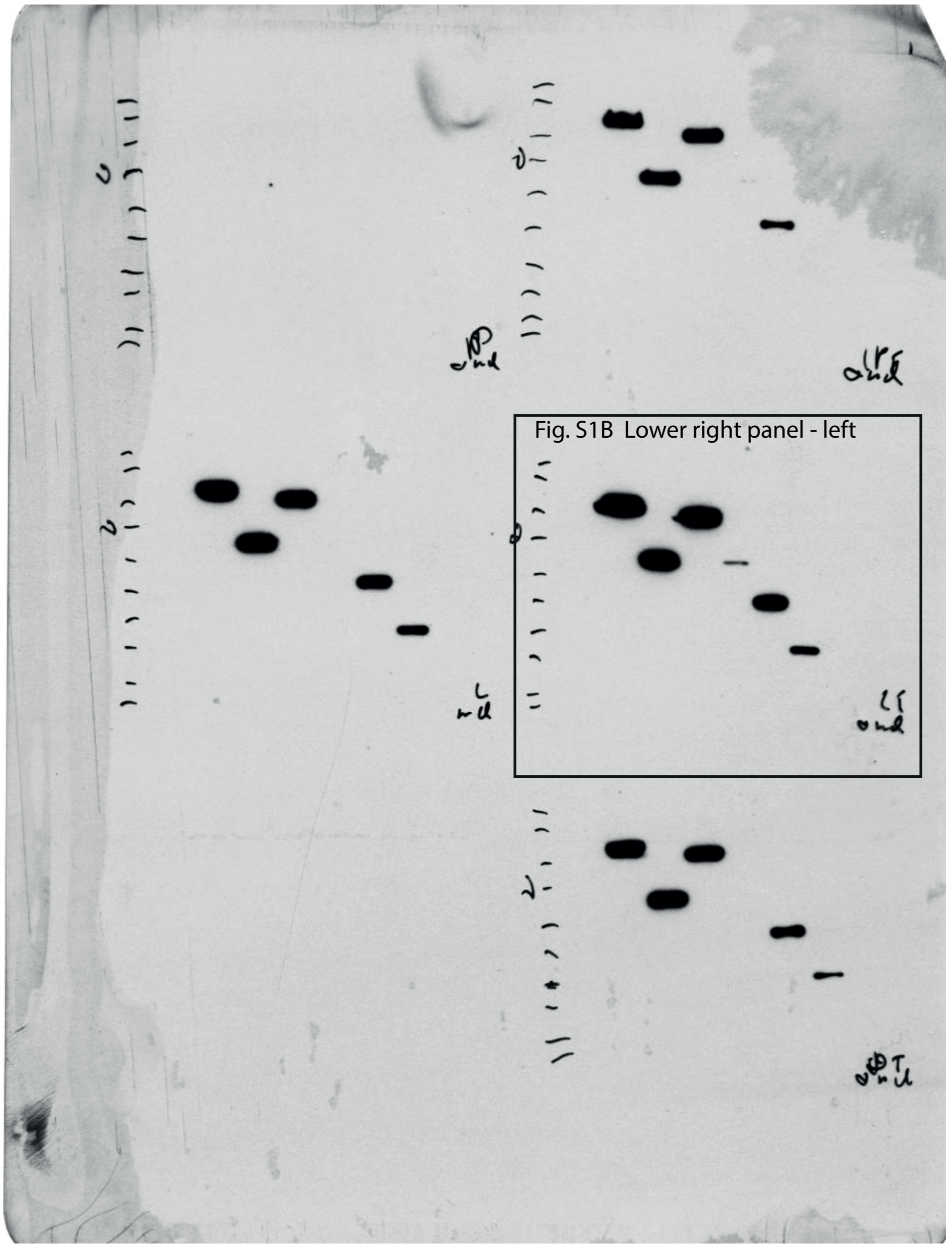
Fig. S1B Lower right panel - left

Fig. S5A

923 bp

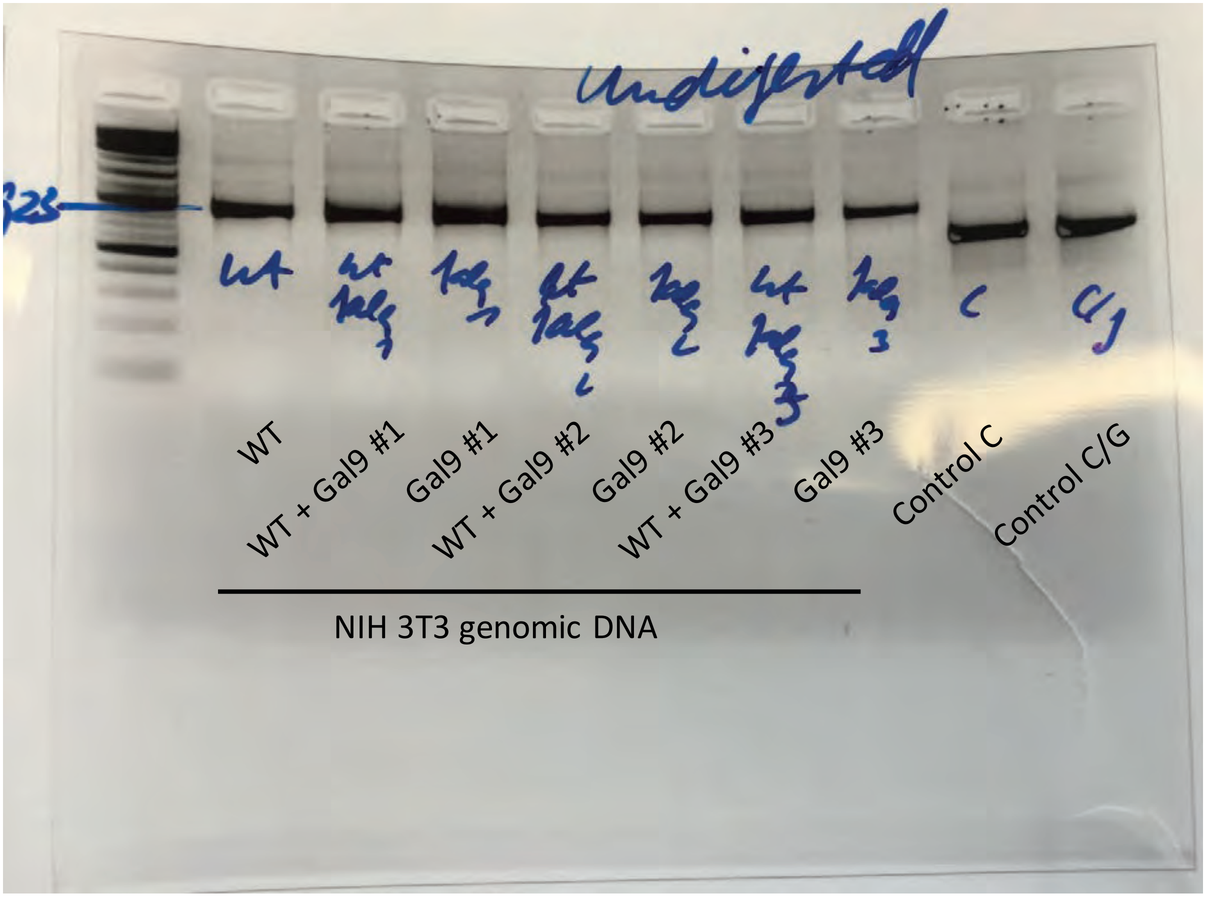

undigested

923 bp
747 bp
176 bp

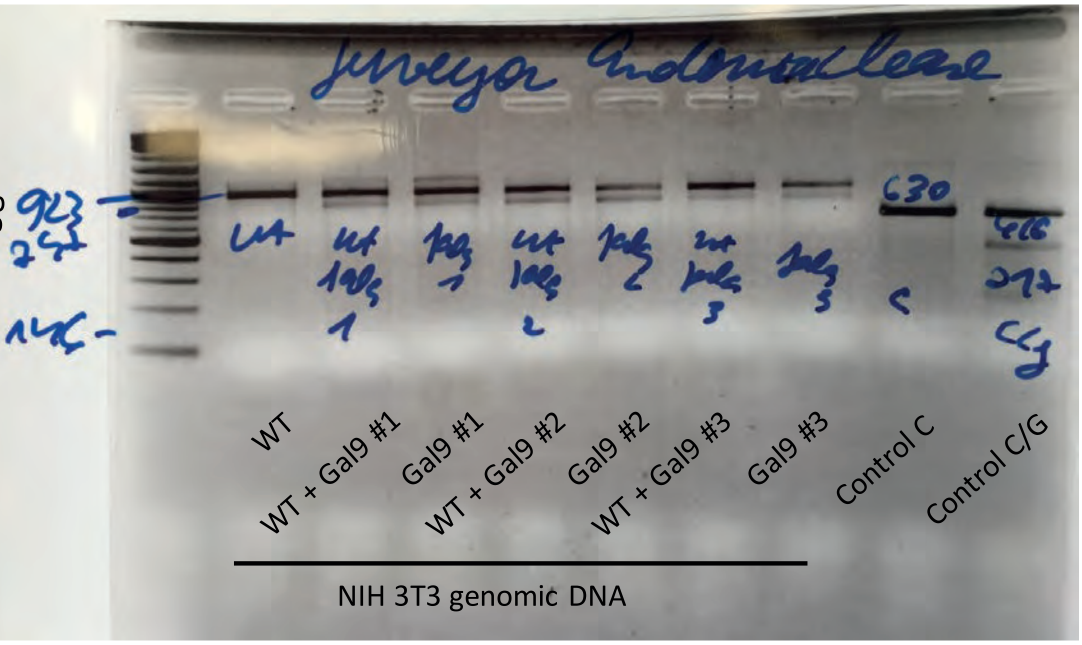

Surveyor endonuclease treated

Fig. S5C upper panel

Fig. S5C lower panel

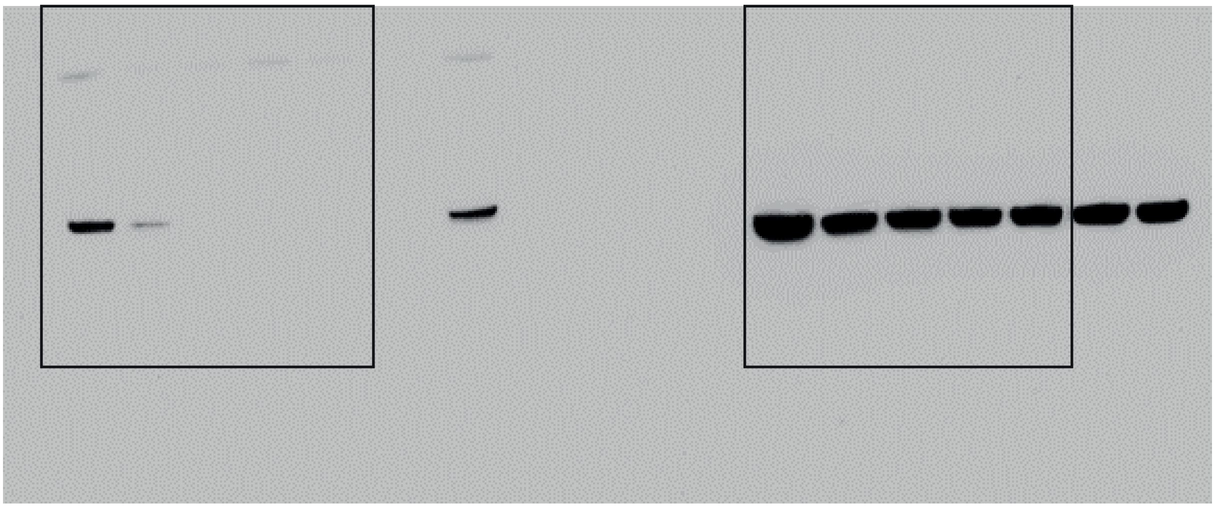

Supplement: S9 Fig — (PDF) [file pone.0316209.s009.pdf]
